# Physiological response of North China red elder container seedlings to inoculation with plant growth-promoting rhizobacteria under drought stress

**DOI:** 10.1371/journal.pone.0226624

**Published:** 2019-12-18

**Authors:** FangChun Liu, HaiLin Ma, ZhenYu Du, BingYao Ma, XingHong Liu, Lin Peng, WenXin Zhang

**Affiliations:** 1 Institute of Resource and Environment, Shandong Academy of Forestry, Jinan, Shandong, China; 2 Shandong Engineering Research Center for Ecological Restoration of Forest Vegetation, Jinan, Shandong, China; University of Salento, ITALY

## Abstract

The issue of how to alleviate the negative effects imposed by water stress is an interesting problem. Plant growth-promoting rhizobacteria (PGPR) colonize the rhizosphere of plants and are known to promote the growth of crops. However, there are few studies characterizing the physiological response of plants to drought stress after PGPR inoculation. The aim of this study was to investigate the effectiveness of different PGPRs in arid environments and then investigated the effects of PGPR inoculation under drought stress on the physiological characteristics and growth of North China red elder (*Sambucus williamsii*) nursery container seedlings. The viable count of different PGPRs under drought stress varies widely, and the drought tolerance of *Acinetobacter calcoaceticus* X128 was significantly higher than that of other PGPRs. In comparison with non-inoculation, inoculation with X128 in an arid environment significantly increased stomatal conductance and mitigated the inhibition of net photosynthetic rate caused by drought stress; this mitigating effect of inoculation is enhanced as the level of drought stress increases. Relative to non-inoculated seedlings, cytokinin levels in the leaves increased by 91.17% under severe drought stress conditions in inoculated seedlings. However, X128 inoculation decreased this deficit to only 44.54%. Compared with non-inoculated seedlings, the relative water content of inoculated seedlings under severe drought stress increased by 15.06%, however the relative conductivity decreased by 12.48%. Consequently, X128 could increase dry matter accumulation of *S*. *williamsii* regardless of watering status, indicative of the greater benefits of PGPR on shoot growth than root. Therefore, inoculation of *A*. *calcoaceticus* X128 under drought conditions play a significant role for alleviating the negative effects imposed by water stress and promoting plant growth.

## Introduction

Drought stress is among the most destructive abiotic stresses that increased in intensity over the past decades affecting world’s food security [1] and may range from moderate and short to extremely severe and prolonged duration, restricting the crop yields [2]. Drought is expected to cause serious plant growth problems for more than 50% of the arable lands by 2050 [3–4]. Plants growing in arid lands or regions facing prolonged abiotic stresses such as water limitation and salt accumulation have also developed specific physiological and molecular stress responses allowing them to thrive under normally unfavorable conditions [5–6]. Drought tolerance or avoidance requires the coordination of morphological and physiological traits [7] and one of the most important and the most immediate physiological mechanisms for regulating plant water loss is stomatal closure [8]. Furthermore, studies have shown that stomatal closure induced by drought can cause photosynthesis to be limited by stomatal or non-stomatal factors, which seriously affects growth and dry matter accumulation [9–11].

At present, the traditional strategies to increase plants' ability of tolerating drought stress involve the use of water-saving irrigation, traditional breeding, and genetic engineering of drought-tolerant transgenic plants [12]. Some new approaches used to improve drought tolerance of plants, including microorganisms, biochars, nanoparticles and some phytohormones also have been available for agriculture [5]. The search for new and efficient microorganisms from unexplored environments to be used in association with plants to alleviate the negative effects imposed by water stress is an interesting alternative [2]. Prudent et al. (2015) reported that soybean is less impacted by water stress when applicated with *Bradyrhizobium japonicum* and thuricin-17 from *Bacillus thuringiensis* [13]. Cohena et al. (2015) reported that *Azospirillum brasilense* could ameliorate the response of *Arabidopsis thaliana* to drought mainly via enhancement of ABA levels [14]. The term ‘plant growth-promoting rhizobacteria’ (PGPR) refers to beneficial microorganisms that live in the rhizosphere of plants and promote plant growth or antagonize pathogens [15–16]. There have many studies on PGPR, which have mainly focused on promoting plant growth [17–18], activating soil nutrients [19–20], reducing fertilizer application [21], and improving plant-induced systemic resistance [22–23]. PGPRs as elicitors of tolerance to abiotic stresses, especially drought stress has been attracted more attention in recent years [1, 12, 24]. Timmusk and Wagner (1999) were the first to show that inoculation of *Paenibacillus polymyxa* confers drought tolerance in *Arabidopsis thaliana* through the induction of the drought-responsive gene ERD15 [25]. Studies have shown that inoculation of *Bacillus amyloliquefaciens* 5113 under drought stress can promote dry matter accumulation in wheat and increase wheat yield [4]. A study by Mayaka et al. (2004) found that *Achromobacter piechaudii* ARV8 producing ACC-deaminase could not inhibit the decrease in the relative water content of tomato and pepper leaves under drought stress, but did promote the rapid recovery of relative water content after rehydration [26]. These studies have shown that the action of PGPR is close ly associated with plant drought.

Cytokinin, namely Kinetin, Trans-zeatin, and zeatin riboside etc. is one of the major mediators of physiological responses throughout plant life span. Therefore, a proper homeostasis is maintained by regulation of their active levels. Physiologically, cytokinin active levels decline in senescing organs under abiotic stresses conditions, providing a signal to nutrients which shows that a shift to reproductive tissues has begun [27–29]. The production of phytohormoness, especially cytokinin, is one of the important mechanisms by which PGPR promotes plant growth [17–18]. Therefore, whether artificially enriching plants with cytokinin by inoculating them with PGPRs could confer drought resistance on plants is an interesting issue. However, considerably less information is available on the characteristics of the physiological response of plants inoculated with phytohormone-producing PGPR under different drought stress intensities. The North China red elder (*Sambucus williamsii*) is a small woody plant in the honeysuckle family and is an important source of oils. It has strong adaptability and is tolerant of a range of soil conditions [30–31]. Container seedlings have largely replaced bare root seedlings in forest nursery production and have become increasingly important because of their benefits on the survival rate after seedling transplantation [32]. Our previous studies have shown that PGPR inoculation can be used as a bioenhancer for plant growth and nutrient uptake in forest container seedling nurseries [33]. Considering the beneficial effect of phytohormone-producing PGPRs under drought stress conditions, we investigated the physiological response of *S*. *williamsii* container seedlings to PGPR inoculation to determine the potential for improving the adaptability of plants to arid environments.

## Materials and methods

### Ethics statement

This article does not contain any studies with human participants or animals performed by any of the authors. The bacteria used in plant experiment have saved in the China General Microbiological Culture Collection Center.

### Survival of different PGPRs in nursery substrate under prolonged drought conditions

Seven PGPRs with known beneficial effects on plant growth and phytohormone production, namely *Brevundimonas* sp. X60, *Pseudomonas* sp. X123, *A*. *calcoaceticus* X128, *Bacillus cereus* Z77, *Paenibacillus polymyxa* SC2, *Bacillus subtilis* GE1, and *Enterobacter cloacae* T026 were selected [34]. All the bacteria selected for use in this investigation were isolated and screened from rhizosphere soil, where water is limited and repeated dry periods occur frequently. X60, X123, X128, and Z77 were saved in the China General Microbiological Culture Collection Center as: CGMCC No. 7669, 7668, 7071 and 7070. The 16S RNA gene sequences were submitted to GenBank under Accession Number KC428750, KC428749, KC428751, KC428752, respectively. Nursery substrate was sterilized thrice by autoclaving at 121°C at 15 psi for 15 min. Seven lots of 300 g sterile nursery substrate were inoculated with each of the different PGPRs. The sterile nursery substrate was prepared by thoroughly mixing *Sphagnum* peat (Jinan Luqing Seeding Co., Ltd., Shandong, China), vermiculite, and perlite (3:1:1, v/v). The effective viable count was 1 × 10^8^ CFU g^-1^. In addition, sterile water was added to adjust the water content of the nursery substrate to 150%. Another lot (300 g) of sterile nursery substrate was not inoculated with any of the PGPRs and watered in the same way as the inoculated ones. The nursery substrates inoculated with different PGPRs were placed in a sterilized tray and naturally dried on a sterile environment; the experiment was repeated three times. The inoculated substrate (including the control) was placed in a sterilized tray and naturally dried in super-clean workbench. After inoculation, 5 g inoculated nursery substrate (including the control) was collected every day, and the number of viable bacteria in nursery substrate was determined until day 26 for each PGPR using the dilution plate-count technique at 28°C for 3 days.

### In plant experiments

#### Seedlings, PGPR and soil

Experiments were performed between March and July 2018 at the Shandong Academy of Forestry nurseries in Jinan, Shandong (36.68°N, 117.07°E). X128 with high cytokinin production was selected for use in this investigation. The indole acetic acid, kinetin, and *trans*-zeati, contents produced by the X128 were 26.31μg·mL^-1^, 310.77 ng·mL^-1^ and 368.73 ng·mL^-1^ respectively, as determined by ultra-performance liquid chromatography–electrospray ionization tandem mass spectrometry [35]. The test plants were 1-year-old *S*. *williamsii* non-woven container seedlings cultivated by the Shandong Forestry Research Institute. The cultivation substrate was peat:perlite:vermiculite = 3:1:1. The average diameter of the 144 *S*. *williamsii* container seedlings was 2.38 ± 0.08 mm, and the mean plant height was 8.51 ± 0.25 cm. The test soil was fluvo-aquic soil with a pH of 7.82. Alkaline hydrolysable nitrogen, available phosphorus, available potassium, and organic matter content were 32.29 mg·kg^-1^, 14.26 mg·kg^-1^, 83.79 mg·kg^-1^ and 15.99 mg·kg^-1^, respectively. The field water holding capacity was 32.13%.

#### Experimental design and measurement methods

Pot experiments were conducted using a randomized block design. On March 26, 2018, a total of 144 pre-cultured *S*. *williamsii* container seedlings were planted in 11.2 kg of soil per pot, with one plant per pot. Before the *S*. *williamsii* seedlings were planted in the pot, they were placed on fine sand plates together with the seedling nursery container (the fine sand plate was covered with filter paper). 25 mL of X128 bacterial solution was diluted to 250 mL. Dilute inoculum or distilled water was added to the fine sand plate, and the inoculum or distilled water were adsorbed into the seedling substrate by capillary action until saturated to water holding capacity. After *S*. *williamsii* Hance container seedlings were planted, they were uniformly tended, and soil moisture content was maintained at 70%–75% of field moisture capacity. On June 10, 2018, drought stress experiments of *A*. *calcoaceticus* X128 inoculation and non-inoculation seedlings were performed by natural drought methods. The design of drought experiments included a well-watered control (WW, relative soil water content 70–75%), light drought (LD, relative soil water content 60–65%), moderate drought (MD, relative soil water content 50–55%), and severe drought (SD, relative soil water content 40–45%). Each treatment contains three repeating groups (six seedlings for each repeating group). The moisture content of the growth media was monitored daily (9:00–10:00 am) using a portable moisture meter (model HH2, Delta-t Devices LTD, Burwell, UK) with a wet sensor (Type Wet-2-K1, Delta-t Devices LTD, Burwell, UK). If the water content was lower than the lower limit of the experimental treatment, the plant was watered to the upper limit of treatment in order to always maintain soil moisture content within the range of the drought design. After 30 days of stress, the morphological differences among different treatments were observed, and the leaves under severe drought stress treatments began to wither, and therefore, the aerial and underground parts of the plants were collected, and relevant physiological indices were determined.

At 10:00–10:30 am on July 11, 2018, the net photosynthetic rate (P_n_), stomatal conductance (g_s_) and intercellular carbon dioxide concentration of leaves (C_i_) were measured using the LI-6400 portable photosynthesis system (LI-COR, USA). Three mature leaves on the sunward side of each seedling were measured. At the end of the experiment, all the seedlings were harvested and fresh and dry weights of the entire shoots and roots after their biochemical parameters were measured. The shoots and roots of one repeating group (six seedling samples) were separated and rinsed with deionized water, and the dry weight was recorded after oven drying at 65 ± 1°C for 24 h (when constant weight was achieved). Relative water content (RWC), relative conductivity, cytokinin content, and abscisic acid (ABA) content were measured, respectively. All the data were recorded from three repeating groups (six seedlings for each repeating group) of each treatment. Cytokinin and ABA contents in *S*. *williamsii* leaves and roots were determined using high performance liquid chromatography (HPLC) using the Agilent HP 1100 series liquid chromatograph (USA) with a UV detection wavelength of 254 nm and a C18 column (250 mm × 4.6 mm, 5 μm) [23]. RWC was determined using the last expanded leaf according to the method described by Barrs and Weatherley (1962) [36], based on the following formula: RWC% = (fresh weight-dry weight) × 100/(turgor weight-dry weight). Relative conductivity was performed using the method described by Liu (2013) [23].

### Statistical analysis

The experiment followed a full factorial (4×2) design with 4 water status (WW, LD, MD and SD) and 2 PGPR inoculations (non-inoculated and inoculated), and 3 replicates for each treatment. Analyses of variance (ANOVA) was performed all data, and the means were compared using the least significant difference (LSD) test (at *P* ≤ 0.05) in SPSS software (version 19.0; SPSS Inc., Chicago, Illinois).

## Results

### Efficacy of PGPR at different drought durations

**Fig 1:**
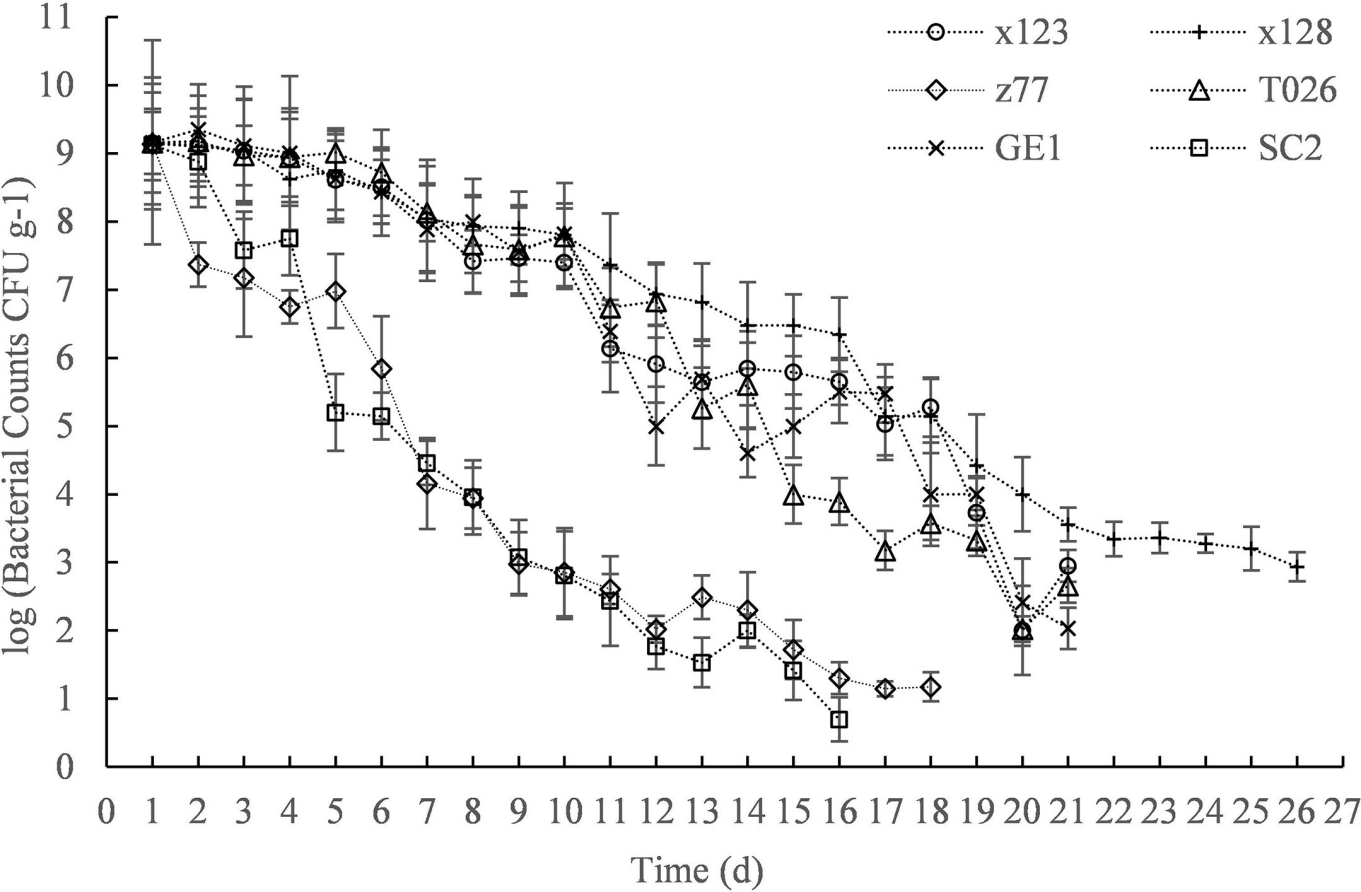
Efficacy of different PGPR in peat at different durations.

The efficacy of different PGPRs in the nursery substrate varied greatly under continuous drought conditions are shown in Fig 1. Assuming that the difference between the initial effective viable counts was not significant (*P* = 0.37), the effective viable counts of the seven PGPRs continued to decrease with increasing drought time. The effective viable counts of SC2, Z77, and T026 (GE3) were nearly 0 at days 19, 21, and 24 of drought, respectively, and the effective viable count of X128 in the nursery substrate was still 417 CFU g^-1^ on day 25 of drought stress. This shows that different PGPRs have very different drought resistances, and that the drought tolerance of X128 is higher than the drought tolerance of the other strains.

### Effect of PGPRs on photosynthetic characteristics under drought stress

ANOVA indicated that drought stress and X128 inoculation significantly affected the P_n_ and *g*_s_ of *S*. *williamsii* seedlings (Table 1). The changes in P_n_ and *g*_s_ of the *S*. *williamsii* container seedlings were generally the same during the test period (Table 2). As stress intensity increased, P_n_ and g_s_ for each treatment exhibited a downward trend. Compared with the corresponding well-watered seedlings, P_n_ decreased by 22.16% and g_s_ decreased by 39.02% for the non-inoculated seedlings under severe drought stress, and P_n_ decreased by 14.40% and g_s_ decreased by 25.53% for the inoculated seedlings under severe drought stress. C_i_ exhibited different patterns of changes under different drought stress intensities. In non-inoculated seedlings, it was lowest under light stress and then increased. In inoculated treatment, it was lowest under moderate stress and increased under severe stress. This analysis showed that the *S*. *williamsii* container seedlings experienced some level of drought stress under the drought intensities used in this study, and *A*. *calcoaceticus* X128 inoculation could not change the tendency of photosynthetic capacity in *S*. *williamsii* to decrease.

**Table 1:** Results of ANOVA showing the F-ratio and statistical significance of the effects of inoculation, drought and their interaction with the effects on hormone content, physical–biochemical characteristics, and growth of *S*. *williamsii* container seedlings.

Source of variation	*P*_n_	*g*_s_	C_i_	Leaf CTK	Root CTKs	Leaf ABA	Root ABA	RWC	RC	ADW	UDW
P	22.98**	37.65**	3.30	196.21**	0.44	14.16**	164.6**	15.64**	16.74**	29.01**	14.16**
D	19.67**	29.17**	13.46*	96.75**	17.94**	64.22**	6.94**	17.14**	24.24**	15.97**	6.25**
D × P	0.65	0.64	4.71*	0.56	3.50*	1.92	12.10**	1.80	3.53*	2.24	0.60

P: PGPR; D: Drought; RWC: Relative water content; RC: Relative conductivity; ADW: Aerial parts dry weight; UDW: Underground parts dry weight; Significance (*P* level)

* *P* < 0.05

** *P <* 0.01.

**Table 2:** Effect of different experimental treatments on photosynthetic characteristics of *S*. *williamsii* under drought stress.

Dry treatments	Photosynthetic rate μmol m^-2^ s^-1^		Stomatal conductance mmol m^-2^ s^-1^		Intercellular CO_2_ concentration μmol m^-2^ s^-1^	
	non-inoculated	inoculated	non-inoculated	inoculated	non-inoculated	inoculated
WW^④^	11.01±0.72^①^a^②^B^③^	11.67±0.34aA	0.41±0.02aB	0.47±0.01aA	267.44±5.97aA	271.53±6.57aA
LD	10.82±0.38aB	11.65±0.27aA	0.39±0.01aB	0.45±0.01aA	223.16±5.62cB	254.95±6.27bA
MD	9.86±0.25bB	11.06±0.20abA	0.32±0.01bB	0.39±0.02bA	242.74±5.34bA	231.67±5.32cA
SD	8.57±0.23cB	9.99±0.36bA	0.25±0.01cB	0.35±0.01bA	251.63±5.80abA	256.67±6.10bA

^①^Numbers are standard errors.

^②^Different small letters indicate significant differences among drought treatments at *P* < 0.05 by LSD.

^③^Different big letters indicate significant differences between non-inoculated and inoculated treatments at *P* < 0.05 by LSD.

^④^WW: well-watered control; LD: light drought; MD: moderate drought; SD: severe drought.

Compared to the non-inoculated seedlings, X128 treatment with the different watering treatments increased P_n_ by 6.00% (WW), 7.68% (LD), 11.87% (MD) and 16.57% (SD), and increased g_s_ by 14.63% (WW), 15.39% (LD), 21.88% (MD) and 40.00% (SD). The increase in photosynthetic rate tended to increase gradually with increasing drought stress. Under the WW treatment, the difference in C_i_ between non-inoculated and inoculated seedlings was not significant (*P* = 0.29), and both decreased to some extent under the LD treatment. Under the MD treatment, the C_i_ of the non-inoculated seedlings began to increase, while it continued to decrease with the inoculated seedlings. Under the SD treatment, the C_i_ of the inoculated seedlings also began to increase significantly (*P* = 0.021). Therefore, X128 inoculation under drought stress can significantly improve the photosynthetic capacity of *S*. *williamsii* leaves and has an impact on its environmental adaptability (Table 1).

### Effect of PGPRs on cytokinin and ABA under drought stress

With normal watering, cytokinin content in leaves of inoculated seedlings increased by 48.43% compared to non-inoculated seedlings, and the difference in cytokinin content in the root system was small (Table 3). As drought stress intensity increased, cytokinin content in the leaves decreased gradually regardless of X128 inoculation status. Compared with the corresponding well-watered seedlings, cytokinin content in the leaves decreased by 7.25% (LD), 29.76% (MD) and 57.35% (SD) in non-inoculated seedlings, and decreased by 6.86% (LD), 27.70% (MD) and 44.54% (SD) in inoculated seedlings. Nevertheless, compared to non-inoculated seedlings, cytokinin levels in the leaves increased by 42.03% (LD), 45.58% (MD), and 91.17% (SD) in inoculated seedlings. Cytokinin level in the root system significantly decreased by 11.42% (inoculated, *P* = 0.027) and by 26.67% (non-inoculated, *P* = 0.014) relative to their respective well-watered control under the SD treatment (*P*<0.05), but the difference between non-inoculated and inoculated seedlings was not significant (*P* = 0.16).

**Table 3:** Effect of different experimental treatments on cytokinin and ABA content in *S*. *williamsii* under drought stress.

Dry treatments	Cytokinin				Abscisic acid			
	Leaf		Root		Leaf		Root	
	non-inoculated	inoculated	non-inoculated	inoculated	non-inoculated	inoculated	non-inoculated	inoculated
WW^④^	10.62±0.30^①^a^②^B^③^	15.02±0.71aA	4.95±0.13aA	4.38±0.10aA	12.34±0.58cA	12.90±0.58bA	6.56±0.23aB	11.16±0.45aA
LD	9.85±0.27aB	13.99±0.58abA	4.43±0.13abA	4.45±0.12aA	12.17±0.57cB	13.11±0.60bA	7.32±0.24aB	10.65±0.40aA
MD	7.46±0.17bB	10.86±0.51bA	4.06±0.11abA	4.11±0.11abA	15.91±0.60bB	17.60±0.70abA	7.09±0.25aB	10.01±0.20abA
SD	4.53±0.17cB	8.66±0.37cA	3.63±0.12bA	3.88±0.15bA	18.01±0.55aB	21.24±0.67aA	7.26±0.19a A	8.06±0.28b A

^①^Numbers are standard errors.

^②^Different small letters indicate significant differences among drought treatments at *P* < 0.05 by LSD.

^③^Different big letters indicate significant differences between non-inoculated and inoculated treatments at *P* < 0.05 by LSD.

^④^WW: well-watered control; LD: light drought; MD: moderate drought; SD: severe drought.

Compared with non-inoculated seedlings, the effect of X128 inoculation on ABA content in the leaves was not significant under the WW treatment (*P* = 0.26), but the ABA content in the root system increased significantly by 70.12% (*P* = 0.004). As drought stress intensity increased, ABA content in the leaves increased gradually regardless of X128 inoculation status. Compared with the WW treatment, ABA content in the leaves increased by 28.93% (MD) and 45.95% (SD) in non-inoculated seedlings, and increased by 36.43% (MD) and 65.43% (SD) in inoculated seedlings. Drought stress had a smaller effect on ABA content in non-inoculated seedlings. In inoculated seedlings, ABA content in the root system decreased as drought stress increased. Under the SD treatment, ABA content was significantly reduced by 27.78% compared to the WW treatment (*P* = 0.016).

### Effect of PGPRs on relative water content and relative conductivity under drought stress

**Fig 2:**
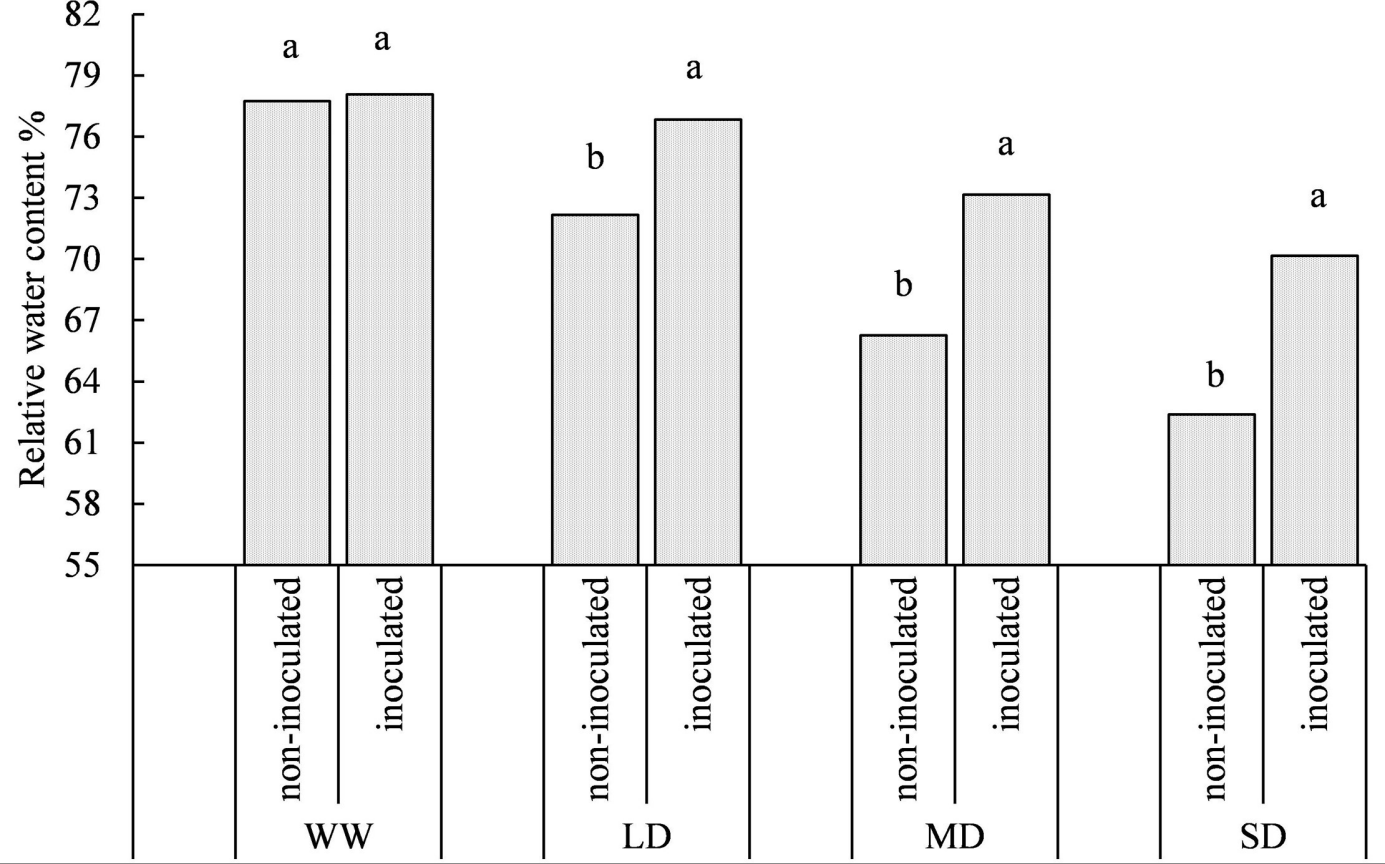
Effect of different treatments on relative water content of *S*. *williamsii*. Different letters indicate significant differences between non-inoculated and inoculated treatments under the same water status.

**Fig 3:**
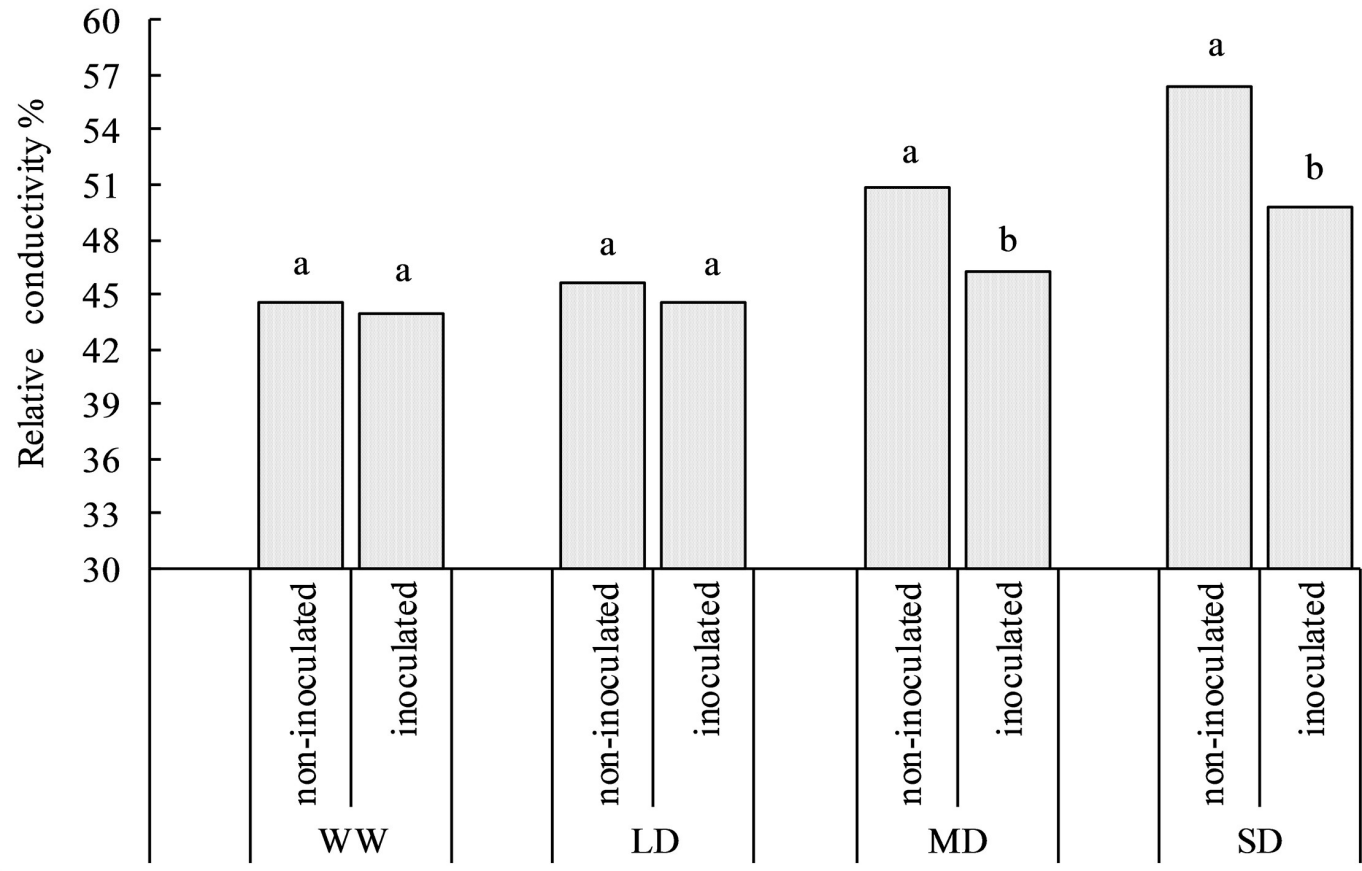
Effect of different treatments on relative conductivity of *S*. *williamsii*. Different letters indicate significant differences between non-inoculated and inoculated treatments under the same water status.

ANOVA indicated that drought stress and X128 inoculation significantly affected RWC of *S*. *williamsii* (Table 1). The RWC of *S*. *williamsii* leaves gradually decreased with increasing drought stress intensity (Fig 2). In the non-inoculated seedlings, the RWC began to decrease under the LD treatment. In the inoculated seedlings, there was no significant difference between the WW and LD treatments (*P* = 0.31). Under the MD treatment, the RWC of the leaves decreased significantly (*P* = 0.022). Under the SD treatment, the RWC of the non-inoculated and inoculated leaves decreased by 19.78% and 10.13% respectively compared to the respective WW treatments. Similar to the change in the RWC, the relative conductivity of the leaves did not differ significantly from the control under the LD treatment regardless of X128 inoculation status (Fig 3). Under the MD treatment, the relative conductivity of leaves of non-inoculated seedlings began to increase, whereas under the SD treatment, the relative conductivity was significantly higher in the inoculated seedlings than in the WW treatment (*P* = 0.012). Under the SD treatment, the relative conductivity of *S*. *williamsii* leaves increased by 28.47% (non-inoculated) and 13.44% (inoculated) compared with the WW treatment. This analysis shows that X128 inoculation in arid habitats cannot change the RWC and relative conductivity of the leaves but can effectively reduce the magnitude of the changes.

### Effect of PGPRs on Dry matter accumulation under drought stress

**Fig 4:**
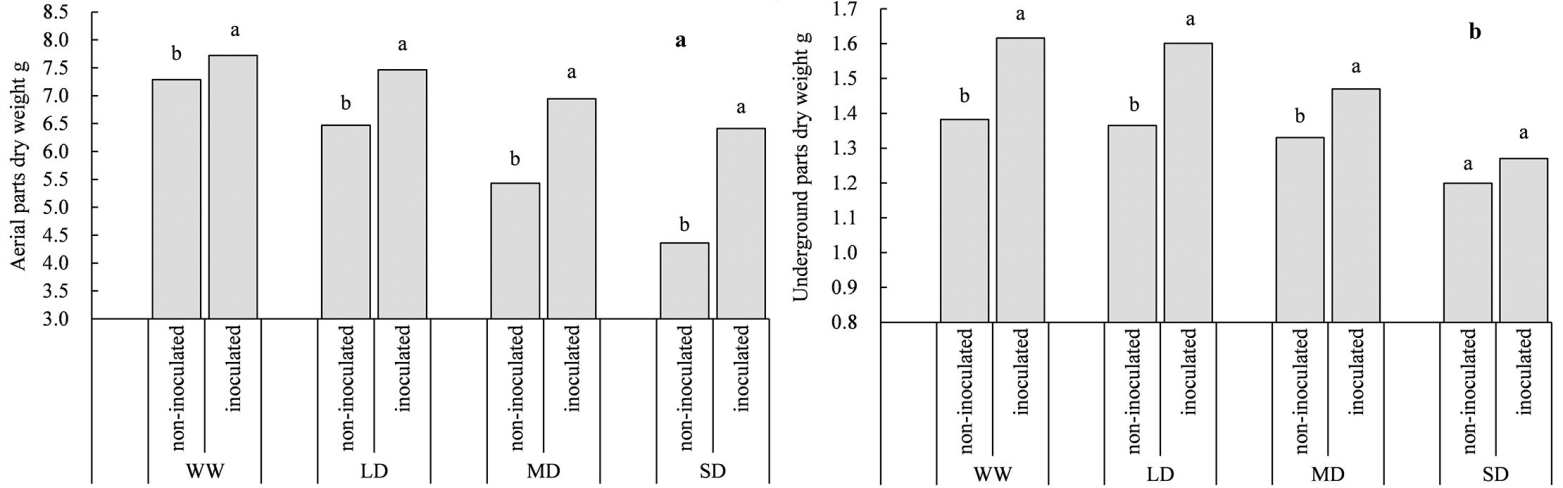
Effect of different treatments on dry matter accumulation of *S*. *williamsii*. Different letters indicate significant differences between non-inoculated and inoculated treatments under the same water status.

Different drought intensities significantly affected dry matter accumulation in the aerial and underground parts of *S*. *williamsii* (Table 1). As drought intensity increased, dry matter accumulation of the aerial (Fig 4A) and underground parts (Fig 4B) of *S*. *williamsii* gradually decreased with or without X128 inoculation. Compared to the WW treatment, dry matter accumulation of the aerial and underground parts decreased by 17.10% and 20.17%, respectively, in the inoculated seedlings under the SD treatment and decreased by 40.19% and 13.24%, respectively, in the corresponding non-inoculated seedlings. The inoculated seedlings increased dry matter accumulation of *S*. *williamsii* to varying degrees regardless of watering status. In the four watering treatments, dry matter accumulation in the aerial parts of inoculated seedlings increased by 7.66% (WW), 15.76% (LD), 24.48 (MD) and 38.51% (SD), and the dry matter accumulation in the underground parts increased by 16.93% (WW), 17.28% (LD), 10.53% (MD), and 7.59% (SD) compared to the non-inoculated seedlings. As drought stress intensity increased, the effect on the root system becomes progressively smaller and the effect on dry matter accumulation in the aerial parts becomes progressively larger. In addition, the root–shoot ratios with the four watering treatments were 18.96 (WW), 21.09 (LD), 24.49 (MD), and 27.50 (SD) in the non-inoculated seedlings, i.e., as drought stress intensity increased, the root–shoot ratio gradually increased; the root–shoot ratio did not differ very much in the inoculated seedlings, at 20.93 (WW), 21.50 (LD), 21.17 (MD), and 20.12 (SD).

## Discussion

Sheik et al. (2011) indicate that ecosystem water budget regulates the abundance and diversity of microbial populations and that rainfall timing is critical at the onset of drought for sustaining microbial populations [37]. Studies have also suggested that inoculation of microorganisms in soil could mitigate plant damage due to drought and improve the drought resistance of turf grasses and promote their growth [38]. These studies have shown that drought has a greater impact on soil microbes, and inoculation of some microorganisms can improve the drought resistance of plants. In the present study, *S*. *williamsii* seedlings were inoculated with different bacteria, and it was found that the bioavailability of different bacteria changed greatly under non-continuous drought conditions, this result confirmed the findings of previous studies. A drought-tolerant PGPR (*A*. *calcoaceticus* X128) was also identified in the present study, and was used in the subsequent experiments presented in this paper.

Drought stress seriously affects plant growth and metabolism, and its effect on photosynthesis is particularly pronounced [39]. Numerous studies have shown that under normal conditions, PGPR can increase photosynthetic rate and promote dry matter accumulation [40]. In the present study, *S*. *williamsii* container seedlings were inoculated with cytokinin-producing PGPRs under conditions of varying drought stress and it was found that although *A*. *calcoaceticus* X128 did not change whether or not P_n_ and g_s_ decreased, it could significantly reduce the magnitude of the decrease; the more severe the drought stress, the greater the effect of X128 inoculation. Factors affecting photosynthesis in plants can be classified as stomatal factors and non-stomatal factors. Stomatal limitation causes C_i_ to decrease, whereas non-stomatal limitation causes C_i_ to increase [41]. The present study found that, both g_s_ and C_i_ were lower in light drought than under normal watering regardless of X128 inoculation status, indicating that the decrease of P_n_ in *S*. *williamsii* leaves was closely associated with stomatal factors. As drought stress intensity increased, C_i_ began to increase significantly as g_s_ decreased in non-inoculated seedlings under moderate drought stress. This indicated that the decrease in P_n_ gradually began to be restricted by non-stomatal factors in addition to stomatal factor restriction, as the photosynthetic activity of mesophyll cells decreased and the structures of the photosynthetic organs suffered some damage. C_i_ began to increase significantly in inoculated seedlings under conditions of severe drought stress, and the decrease in P_n_ began to be limited by non-stomatal factors. Therefore, *S*. *williamsii* inoculated with X128 experienced mild drought stress even under severe drought conditions, and PGPR inoculation in arid environments had a large effect on the drought adaptability of plants. The X128 strain selected in the present study can produce different concentrations and types of cytokinins, which can promote stomatal opening and improve the photosynthetic capacity of plants [42]. The beneficial effect of X128 inoculation on cytokinin content in the leaves may be one of the reasons for improving the photosynthetic rate of *S*. *williamsii* and promoting the growth of plants in arid environments.

It is generally believed that cytokinins are mainly synthesized by roots, and then transported by roots through xylem to stems and leaves to regulate the growth of the aboveground plants of plants [43]. The results of the present study showed that compared to normal watering, drought stress caused a significant decrease in the cytokinin content in *S*. *williamsii* leaves, whereas *A*. *calcoaceticus* X128 inoculation significantly increased cytokinin content in arid environments. The *A*. *calcoaceticus* X128 selected in the present study could produce different concentrations and types of cytokinins. On the one hand, the cytokinins inherently secreted by X128 can be transported to aerial plant tissues through the root system, and on the other hand, they may stimulate the plants to inherently produce higher concentrations of cytokinins, which may be the main reason by which X128 increases cytokinin content in *S*. *williamsii* leaves. Arkhipova et al. (2007) confirmed that cytokinin can increase the plant photosynthetic rate by regulating stomatal opening [44]. Therefore, the significant decrease of cytokinin content in the leaves is one of the possible reasons why drought causes a significant decrease in plant photosynthetic rate. The increase of cytokinin content in the leaves can promote stomatal opening, thereby effectively inhibiting the excessive decrease of the photosynthetic rate in plant leaves in arid environments. Studies have confirmed that ABA acts as a hormonal stress signal in xylem under drought conditions and can be transported from the roots to various parts of the stem to regulate transpiration loss and leaf growth [45]. The results of the present study showed that under normal watering conditions, X128 inoculation promoted a significant increase in ABA content in the roots of *S*. *williamsii* container seedlings, which may be the main reason why Arkhipova et al. (2007) [44] reported that PGPR has some inhibitory effects on plant root growth. In arid habitats, ABA content in the roots of plants inoculated with X128 was significantly reduced, whereas the ABA content in the leaves was significantly increased. Therefore, X128 inoculation in arid habitats might increase the permeability of roots to water or increase the transport of ions into the xylem, promoting the transport of ABA with water from the roots into the leaves, resulting in a decrease in stomatal opening or even complete closure. This analysis shows that *A*. *calcoaceticus* X128 inoculation in arid habitats can regulate stomatal activity by affecting phytohormones content, thereby positively enhancing their drought adaptability and promoting growth.

The analysis presented in this paper shows that *A*. *calcoaceticus* X128 can increase cytokinin content in leaves and promote the transport of ABA produced in the roots to the leaves under drought stress and reduce the damage to *S*. *williamsii* seedlings. However, the mechanisms of action of PGPR that signal the defenses of these plants, such as hormones and auxin, should be the focus of future researches. The present study showed that one of the possible mechanisms by which PGPR alleviate the drought stress and interfere with the suppression of plant growth under drought conditions is the production of cytokinin. Further studies are needed to identify metabolites and enzymes that mediate the signaling and transduction that confer tolerance to drought stress to plants inoculated with PGPR. Additionally, different plants growing in arid lands have developed diversity strategies to cope with drought, spanning from avoidance or migration, to drought tolerance. Although PGPR achieved beneficial effect on *S*. *williamsii* seedlings in this study, which does not mean that PGPR will have the same effect on other plants. Therefore, the application of PGPR in other plants under drought stress is also the focus of future research.

## Conclusions

The results of this study indicate that *A*. *calcoaceticus* X128 inoculation under drought conditions is an effective way to improve the adaptability of plants to arid environments. The stomatal conductance and net photosynthetic rate of *S*. *williamsii* was significantly increased by X128 inoculation in an arid environment. Under drought stress, X128 inoculation significantly increased cytokinin content in leaves and promoted the transport of ABA produced in the roots to the leaves. Consequently, X128 inoculation increased dry matter accumulation in the aerial parts by 38.51%, as well as that in the underground parts by 7.59% under severe drought conditions, respectively. Therefore, inoculation of *A*. *calcoaceticus* X128 under drought conditions has great potential for improving the adaptability of plants to arid environments, reducing the intensity of drought stress, and promoting plant growth.

## Supporting information

S1 File Preservation certificate of *Acinetobacter calcoaceticus* X128. (PDF)

S2 File The 16S RNA gene sequence of *Acinetobacter calcoaceticus* X128. (DOC)

S1 Fig Relative soil water content during natural drought stress. (TIF)
