# Supplementary material for: Physiological response of North China red elder container seedlings to inoculation with plant growth-promoting rhizobacteria under drought stress
Source: PLoS One. 2019 Dec 18;14(12):e0226624. doi: 10.1371/journal.pone.0226624 (PMC6919619; doi:10.1371/journal.pone.0226624)
Supplement: S1 File — (PDF) [file pone.0226624.s001.pdf]

中国微生物菌种保藏管理委员会
普通微生物中心
China General Microbiological Culture Collection Center (CGMCC)

地址：北京市朝阳区北辰西路1号院3号，　中国科学院微生物研究所，　邮政编码：100101
电话：010-64807355　传真：010-64807288　电子邮件：cgmcc@im.ac.cn　http://www.cgmcc.net

受理通知书（收据）
存活性报告书

用于专利程序的微生物保存

发出日期 2012 年 12 月 31 日

(请求保藏人或代理人的姓名、地址)

马海林
山东省林业科学研究院
山东省济南市文化东路42号

本保藏中心登记入册编号

CGMCC No. 7071

申请专利的发明名称

申请号NO.
申请日期　　年　　月　　日

你（们）提供的请求保藏并注明以下鉴定

参据的微生物（株）： X128

上述请求保藏的微生物（株）附有

☐ 科学描述

☑ 建议的分类命名： 乙酸钙不动杆菌

Acinetobacter calcoaceticus

该微生物（株）已于 2012 年 12 月 31 日由本保藏中心收到，并登记入册。

根据你（们）的请求，由该日起保存三十年，在期满前收到提供微生物样品的请求后再延续保存五年。

该微生物（株）的存活性经本保藏中心于 2012 年 12 月 31 日检测，结果是

（1）存活 √　　　　（2）失活 /

中国微生物菌种保藏管理委员会普通微生物中心负责人签字和日期

姓名 [signature]　　2013 年 01 月 15 日

中国微生物菌种保藏管理委员会普通微生物中心
