# Supplementary material for: Physiological response of North China red elder container seedlings to inoculation with plant growth-promoting rhizobacteria under drought stress
Source: PLoS One. 2019 Dec 18;14(12):e0226624. doi: 10.1371/journal.pone.0226624 (PMC6919619; doi:10.1371/journal.pone.0226624)
Supplement: S2 File — (DOC) [file pone.0226624.s002.doc]

acttgggggg aggctttaca catgcaagtc gagcggagag aggtagcttg ctactgatct 60

tagcggcgga cgggtgagta atgcttagga atctgcctat tagtggggga caacatttcg 120

aaaggaatgc taataccgca tacgtcctac gggagaaagc aggggatctt cggaccttgc 180

gctaatagat gagcctaagt cggattagct agttggtggg gtaaaggcct accaaggcga 240

cgatctgtag cgggtctgag aggatgatcc gccacactgg gactgagaca cggcccagac 300

tcctacggga ggcagcagtg gggaatattg gacaatgggc gcaagcctga tccagccatg 360

ccgcgtgtgt gaagaaggcc ttatggttgt aaagcacttt aagcgaggag gaggctactt 420

tagttaatac ctagagatag tggacgttac tcgcagaata agcaccggct aactctgtgc 480

cagcagccgc ggtaatacag agggtgcaag cgttaatcgg atttactggg cgtaaagcgc 540

gcgtaggcgg ctaattaagt caaatgtgaa atccccgagc ttaacttggg aattgcattc 600

gatactggtt agctagagtg tgggagagga tggtagaatt ccaggtgtag cggtgaaatg 660

cgtagagatc tggaggaata ccgatggcga aggcagccat ctggcctaac actgacgctg 720

aggtgcgaaa gcatggggag caaacaggat tagataccct ggtagtccat gccgtaaacg 780

atgtctacta gccgttgggg cctttgaggc tttagtggcg cagctaacgc gataagtaga 840

ccgcctgggg agtacggtcg caagactaaa actcaaatga attgacgggg gcccgcacaa 900

gcggtggagc atgtggttta attcgatgca acgcgaagaa ccttacctgg ccttgacata 960

gtaagaactt tccagagatg gattggtgcc ttcgggaact tacatacagg tgctgcatgg 1020

ctgtcgtcag ctcgtgtcgt gagatgttgg gttaagtccc gcaacgagcg caaccctttt 1080

ccttatttgc cagcgagtaa tgtcgggaac tttaaggata ctgccagtga caaactggag 1140

gaaggcgggg acgacgtcaa gtcatcatgg cccttacggc cagggctaca cacgtgctac 1200

aatggtcggt acaaagggtt gctacctagc gataggatgc taatctcaaa aagccgatcg 1260

tagtccggat tggagtctgc aactcgactc catgaagtcg gaatcgctag taatcgcgga 1320

tcagaatgcc gcggtgaata cgttcccggg ccttgtacac accgcccgtc acaccatggg 1380

agtttgttgc accagaagta gctagcctaa ctgcaaaaga gggcggttac caccggttgg 1440

cccgaataaa agt 1453
