# Supplementary figures and images for: Physiological response of North China red elder container seedlings to inoculation with plant growth-promoting rhizobacteria under drought stress
Source: PLoS One. 2019 Dec 18;14(12):e0226624. doi: 10.1371/journal.pone.0226624 (PMC6919619; doi:10.1371/journal.pone.0226624)

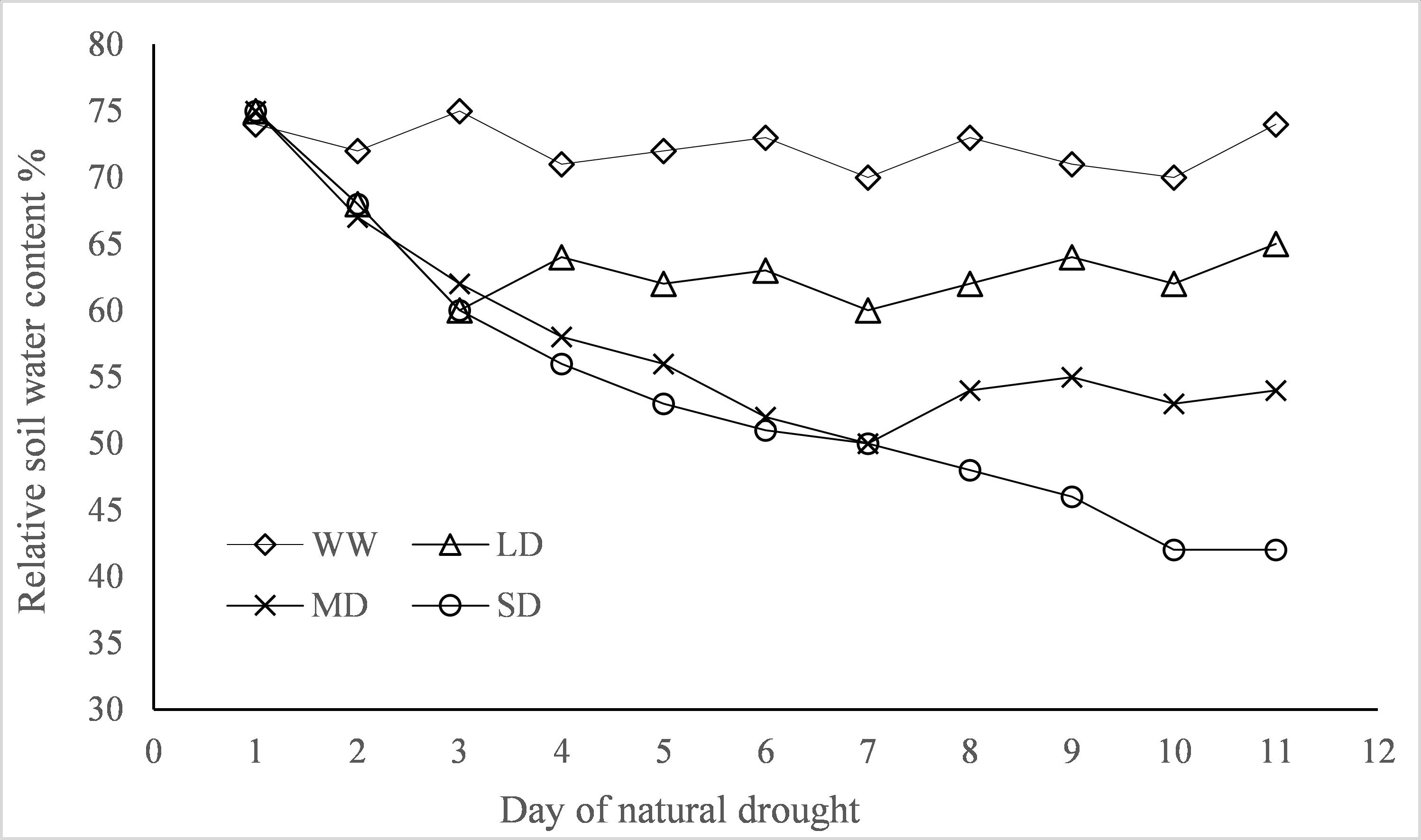

Supplement: S1 Fig — (TIF) [file pone.0226624.s003.tif]
